# Sequencing whole mitochondrial genomes to assess genetic divergence between proposed silver-haired bat (*Lasionycteris noctivagans)* populations

**DOI:** 10.1080/23802359.2020.1841577

**Published:** 2020-12-24

**Authors:** Marissa Monopoli, Jamin G. Wieringa, Juliet Nagel, David M. Nelson, H. Lisle Gibbs

**Affiliations:** a Department of Evolution, Ecology and Organismal Biology, The Ohio State University, Columbus, OH, USA; b Ohio Biodiversity Conservation Partnership, Columbus, OH, USA; c Appalachian Lab, University of Maryland Center for Environmental Science, Frostburg, MD, USA

**Keywords:** *Lasionycteris noctivagans*, chiroptera, mitochondrial genome

## Abstract

The geographic distributions of eastern and western *Lasionycteris noctivagans* populations suggest they could be genetically isolated, but this has rarely been assessed using genetic data. Here, we evaluate this possibility by sequencing the complete mitochondrial genome of four silver-haired bats from eastern and western populations. The three usable mitogenomes were closely associated with other Vespertilionid bats and the phylogenetic tree revealed the two western individuals grouping together to form their own clade. Our results support the idea of small but significant genetic differences between eastern and western populations of these bats, but this should be tested further.

Silver-haired bats *(Lasionycteris noctivagans*) are commonly killed by wind turbines in North America (Arnett and Baerwald 2013) and therefore, the development of genetic biomarkers to source bats could be useful for assessing the geographic magnitude of turbine impacts. Cryan (2003) suggested the possibility of two distinct silver-haired bat populations in the eastern and western parts of their range. This comes from the finding that unique ectoparasites are found on geographically restricted populations (Talbot et al. 2016) but previous studies have not identified genetic distinctions between east and west. Sovic et al. (2016) found support for two populations by observing evidence for two genetic clusters using RADseq data but lacked samples from western populations. In this study, we evaluated the complete mitochondrial genomes of four *L. noctivagans* collected across the US and created a phylogenetic tree to identify possible divergence between hypothesized groups.

Wing tissue was collected from four *L. noctivagans* bats collected in California (40.74, −123.91; MT774149; male; live caught wing punches on 14 Jul 2015), Idaho (42.58, −112.88; MT774150; unknown sex; turbine kill on 19 Jul 2013), Rhode Island (41.78, −71.40; see below; female; found dead in a residential area on 3 Aug 2014), and West Virginia (39.08, −79.42; MT774151; male; turbine kill on 21 May 2015). Qiagen DNeasy blood tissue extraction was performed and was sequenced using Illumina NovaSeq. DNA is currently stored at the Museum of Biological Diversity at The Ohio State University (OSUM MC-29 – MC-32, in order as above; RI specimen at Harvard Museum of Comparative Zoology MCZ_69469). Raw reads were trimmed using Trimmomatic (Bolger et al. 2014), assembled in Norgal (Al-Nakeeb et al. 2017), and annotated via MITOS (Bernt et al. 2013). A cross-species check was performed using BLAST to ensure the accuracy of called genes. Finally, Unipro Ugene was employed to build a phylogenetic tree (Okonechnikov et al. 2012) using PhyML with default settings and 100 bootstraps (Guindon et al. 2010).

The size of the assembled mitogenome was 16,496 base pairs, with each consisting of 22 tRNA, two rRNA, and 13 protein-coding genes. This structure is consistent with other species of Vespertilionid bats and the four sequences were most closely associated with other Vespertilionidae using BLAST. However, BLAST revealed that the Rhode Island bat was closely related to different species of Vespertilionidae compared to the other three. Challenges arose when assembling the mitogenome of this individual; the genome was less complete and had more ambiguous base calls, and could not be uploaded to GenBank due to countless issue. For these reasons, this bat was not included in further analyses. A phylogeny was built using two *Eptesicus serotinus* (Nam et al. 2015) and one *Hypsugo alaschanicus* (Shi et al. 2017) as outgroups (Figure 1). This phylogenetic tree reveals the two western individuals forming their own clade. Overall, these findings support the notion of genetic divergence between an eastern and western subpopulation within this species, with the possibility to assign individuals to different regions. This is supported by Fst values calculated between east and west (0.13, revealing moderate differentiation [Hartl and Clark 1997]). For discerning east vs west, we have identified six possible SNPs unique between the eastern and western individuals: 2 in COI, 2 in COII, and two in the control region (data not shown).

**Figure 1.**
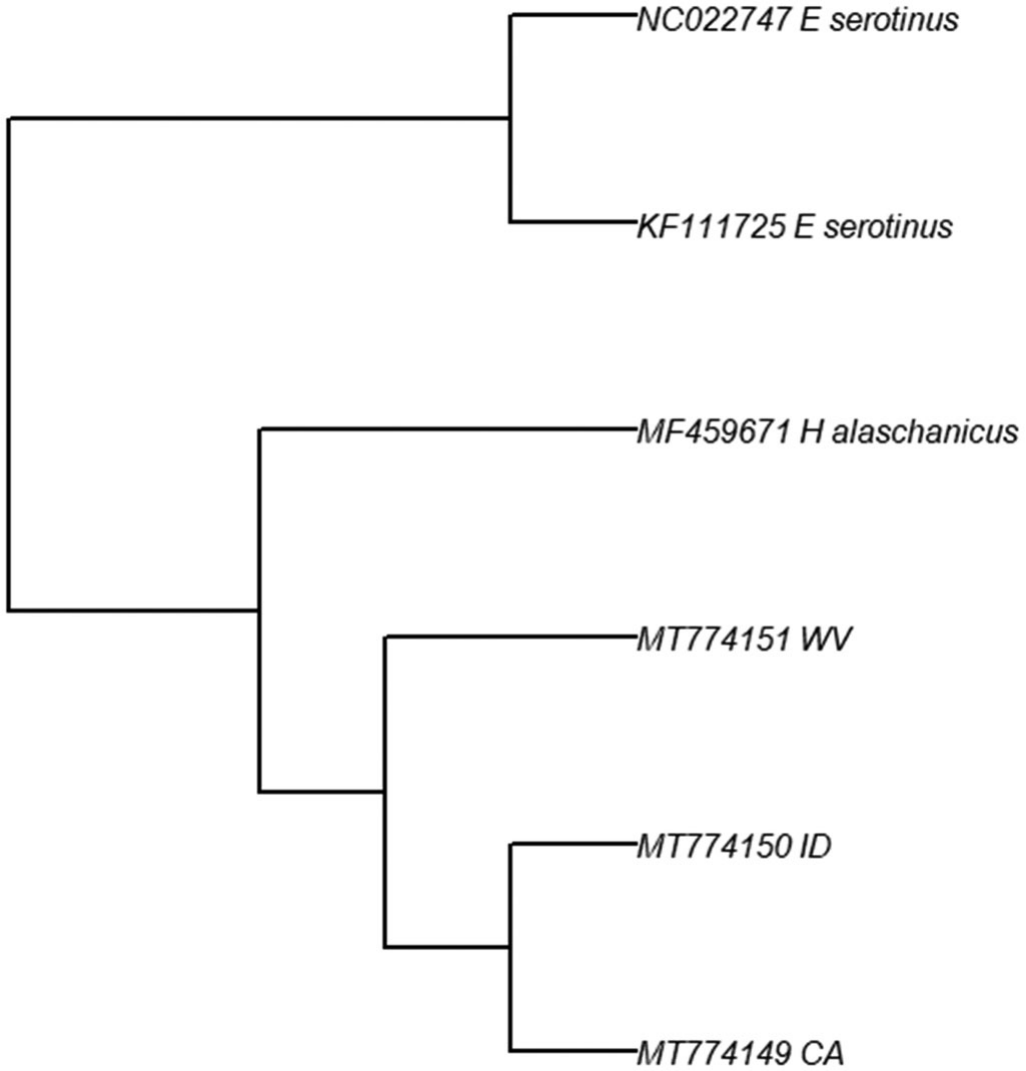
Maximum likelihood phylogeny for three *L. noctivagans* from across their range. The clustering of the two Western individuals (ID and CA) show support for possible structuring across their range.

Since previous studies have not identified population structuring among silver-haired bats using the nuclear genome (although this is being explored further [J Nagel, unpublished results]), one possible explanation for the differentiation is sex-biased migration. Whitaker and Hamilton (1998) state that, while females travel to the wintering range, males remain throughout the year. It has additionally been proposed that the minor “migratory” movements of males are more erratic than traditional north/south movements females follow (Fraser et al. 2017). Due to its matrilineal nature, the mitogenome may reveal differences based on sex-biased migration that the nuclear genome does not. Furthermore, the smaller effective population represented by mitochondria may result in greater sensitivity to genetic divergences. While our results are indicative of some level of an east-west split among *L. noctivagans*, further studies with larger sample sizes are needed to confirm. Future genetic work on *L. noctivagans* may benefit from supplementing nuclear marker techniques with the mitogenome to understand east-west divergence and to source bats killed at wind farms.

## Data Availability

All assembled mitochondrial genomes are available on GenBank using the ascension numbers: MT774149- MT774151. Sequence data that support the findings of this study are available in Data Dryad at https://doi.org/10.5061/dryad.sj3tx962w, Wieringa et al. (2020).
